# Experimental comparison of African swine fever virus exposure through contaminated feed and infected pig organ homogenate

**DOI:** 10.3389/fvets.2026.1830491

**Published:** 2026-07-08

**Authors:** Ara Cho, Ngoc Anh Bui, Vuong Nghia Bui, Duy Tung Dao, Minh Duc Nguyen Do, Hu Suk Lee, Eun-Yeong Bok, Tai-Young Hur, Young-Hun Jung, Han Gyu Lee, Seogjin Kang, Sang-Ik Oh

**Affiliations:** 1 Division of Animal Diseases & Health, National Institute of Animal Science, Rural Development Administration, Wanju, Republic of Korea; 2 Virology Department, National Institute of Veterinary Research, Hanoi, Vietnam; 3 Laboratory of Veterinary Pathology and Biosafety Research Institute, College of Veterinary Medicine, Jeonbuk National University, Iksan, Republic of Korea; 4 College of Veterinary Medicine, Chungnam National University, Daejeon, Republic of Korea; 5 Dairy Science Division, Department of Animal Resource Development, National Institute of Animal Science, Rural Development Administration, Cheonan, Republic of Korea

**Keywords:** African swine fever (ASF), biosecurity, feed contamination, oral ingestion, organ homogenate, swill feed

## Abstract

**Introduction:**

African swine fever virus (ASFV) causes a devastating hemorrhagic disease in swine. Oral exposure through ASFV-contaminated feed or ASFV-infected pork products represents a major transmission pathway; however, the relative risk associated with these two exposure scenarios remains incompletely defined under controlled experimental conditions.

**Methods:**

In this study, we conducted three independent *in vivo* experiments in domestic pigs to compare oral ingestion of genotype II ASFV isolated from Vietnam via contaminated commercial feed (dry and wet forms; 2 × 10^6^ HAD_50_ per pig) and ASFV-infected organ homogenate prepared from spleen and liver (5 × 10^6^ HAD_50_ per pig). Clinical signs were monitored daily for 28 days, and virological testing and gross lesions were assessed according to the predefined sampling and necropsy schedule.

**Results & Discussion:**

None of the feed-exposed pigs (dry feed, *n* = 5; wet feed, *n* = 3) showed clinical signs, viremia, or gross lesions during the experimental period. In contrast, oral administration of ASFV-infected organ homogenate (*n* = 5) resulted in 100% mortality (mean survival: 8.4 ± 1.8 days). Viremia was first detected at 2.8 ± 0.5 days post-infection (dpi), followed by sequential onset of oral (3.2 dpi), nasal (4.0 dpi), and rectal (4.4 dpi) shedding. All organ homogenate-fed pigs exhibited gross lesions consistent with acute ASFV infection, including hemorrhages in the spleen, kidneys, liver/gallbladder, and multiple lymph nodes. Under the conditions tested, exposure to commercial feed experimentally spiked with ASFV did not result in detectable systemic infection, whereas ingestion of infected organ homogenate consistently produced acute ASF. These overall results emphasized the critical importance of both feed safety protocols and continued enforcement of swill-feeding bans and safe disposal of infected carcasses.

## Introduction

African swine fever (ASF) is a highly contagious hemorrhagic disease affecting domestic pigs and wild boar, characterized by high fatality rates and devastating economic consequences for the global swine industry (1, 2). The transcontinental spread of the ASF virus (ASFV) has been linked to two major swill feeding-related introductions from Africa. The first introduction occurred in Portugal in 1957 when contaminated food waste from African sources was fed to pigs near Lisbon, establishing genotype I ASFV circulation in the Iberian Peninsula for over three decades (3, 4). The second introduction occurred in the Republic of Georgia in 2007, when ASFV-contaminated pork products transported by ships from southeastern Africa were fed to pigs through port waste disposal, introducing genotype II ASFV (5, 6). This genotype II ASFV strain subsequently spread across Europe and entered China in 2018, causing rapid dissemination across Asian countries, including Vietnam, the Philippines, and the Republic of Korea (7–9).

Given the limited availability of effective vaccines, understanding transmission pathways is critical for developing effective prevention strategies, particularly because oral exposure is a major recognized ASFV transmission route (1, 9). Oral transmission through contaminated feed and infected pork products poses substantial risks for virus introduction not only to susceptible farms but also to ASF-free countries (10). Although some studies have characterized oral ASFV infection through feed (11, 12), few studies have experimentally compared these oral exposure scenarios within a single study.

Therefore, we designed a controlled experimental comparison of two oral exposure scenarios in domestic pigs to evaluate the oral infection efficiency of ASFV: (i) commercial feed (dry and wet forms) contaminated with ASFV, and (ii) organ homogenate prepared from ASFV-infected porcine organ tissues. Clinical outcomes and detectable systemic infection were evaluated across all exposure groups, and virological kinetics were characterized in pigs exposed to infected organ homogenate. This study aimed to provide evidence-based data to support current biosecurity policies and enhance risk assessment frameworks for ASF prevention in commercial swine operations. Moreover, the results of the present study may also inform quarantine strategies for protecting ASF-free countries.

## Materials and methods

### Study design and overview

**Figure 1:**
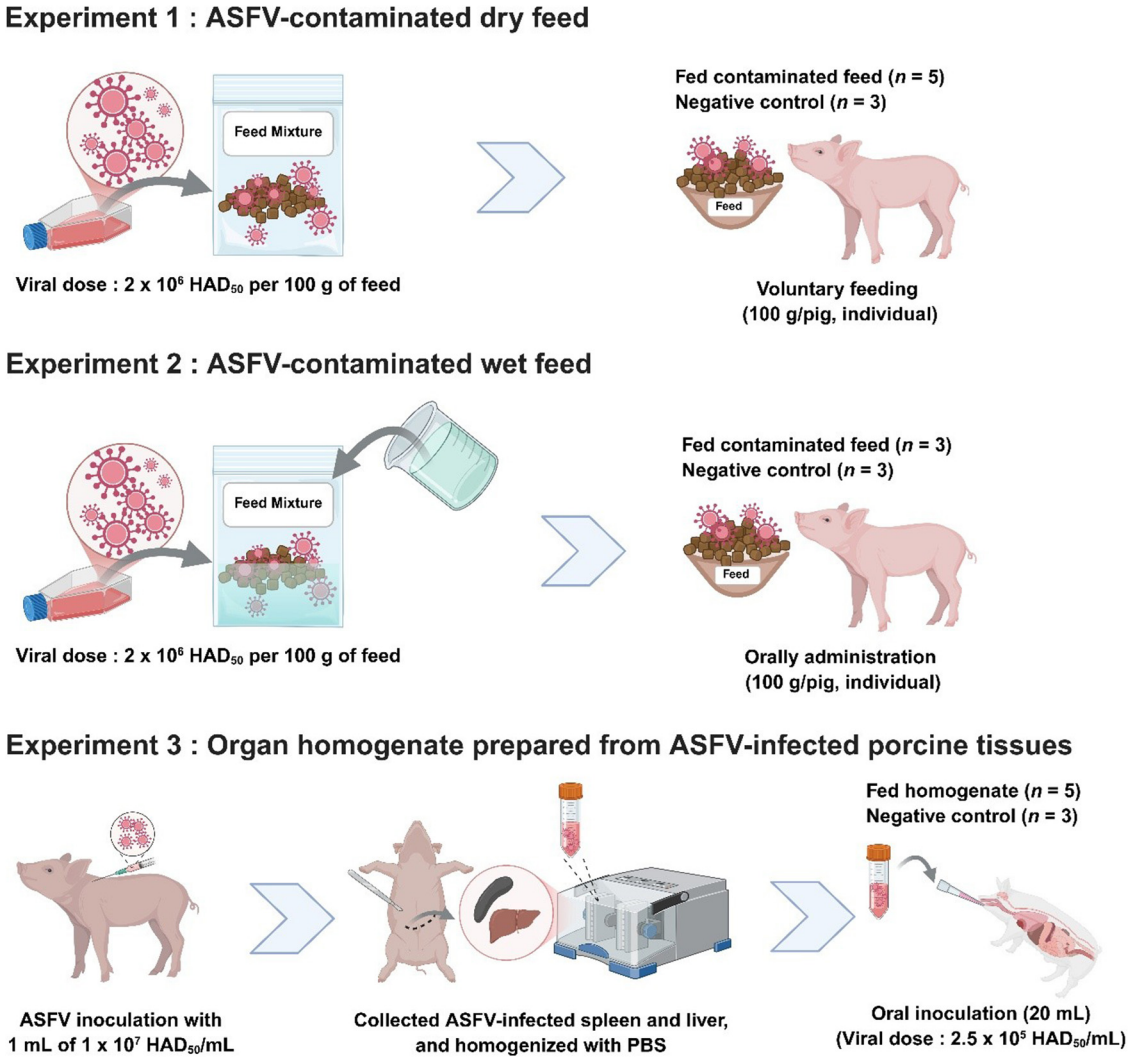
Experimental design of three African swine fever virus exposure trials involving contaminated feed matrices and infected pig organ tissue homogenates.

All experiments were conducted at the enhanced animal biosafety level 2 facility of the National Institute of Veterinary Research (Hanoi, Vietnam) in accordance with the guidelines of the Vietnamese government. The animal experiments were approved by the Institutional Animal Care and Use Committee of the National Institute of Animal Science, Republic of Korea (Approval No. NIAS2022-556). The experimental design consisted of three independent trials to evaluate infection outcomes following three ASFV exposure models: (i) ASFV-contaminated dry feed, (ii) ASFV-contaminated wet feed, and (iii) organ homogenate prepared from ASFV-infected porcine organ tissues (Figure 1). A genotype II ASFV strain isolated from blood of pigs during an ASF outbreak in Thanh Hóa province (Vietnam) in 2020 (GenBank accession no. OP615344) was used throughout all three experiments. This strain has been previously characterized in a pig-to-pig transmission study and recently used to compare their pathogenicity with a recombinant genotype I/II ASFV (13, 14) and was propagated in porcine alveolar macrophages and titrated by the 50% hemadsorbing dose (HAD_50_) assay, as previously described (8).

For each experiment, 6- to 7-week-old Landrace × Yorkshire pigs were randomly allocated to an ASFV-exposed group (*n* = 5 for dry-feed and organ-homogenate trials; *n* = 3 for the wet-feed trial) and a separate negative control group (*n* = 3 per trial). All experimental pigs were confirmed negative for the following endemic pathogens using quantitative polymerase chain reaction (qPCR) and enzyme-linked immunosorbent assay before oral ASFV exposure: foot-and-mouth disease virus, porcine reproductive and respiratory syndrome virus, classical swine fever virus, porcine circovirus 2, and ASFV. Animals were individually housed and acclimated to the facility for 7 days prior to ASFV infection, with commercial feed provided twice daily and water provided *ad libitum*. All virus exposures through contaminated feed or organ homogenate were administered once on day 0, with clinical monitoring continuing for 28 days post-infection (dpi) or until humane endpoints (15) were reached. Clinical monitoring was standardized across all three trials; however, blood and swab sampling differed according to the exposure model and the onset of clinical signs.

### ASFV-contaminated dry- and wet-feed exposure models

Two independent feeding trials were conducted to evaluate the efficiency of ASFV infection through contaminated dry and wet commercial feed. Commercial complete pig feed was used as the exposure matrix in both models. An ASFV inoculum was diluted in phosphate-buffered saline (PBS, pH 7.4) and thoroughly mixed with the feed to achieve a nominal dose of 2 × 10^6^ HAD_50_ per 100 g of feed. The inoculum was added dropwise to the feed and manually mixed in a sterile container until homogeneous, and the contaminated feed was offered to the pigs immediately after preparation. This procedure was designed to simulate extrinsic (post-production) contamination of finished commercial feed and does not address virus introduction through feed ingredients during the manufacturing process.

In the dry-feed exposure model, each pig in the exposed group received 100 g of ASFV-contaminated dry feed in an individual feeding trough on day 0 and voluntarily consumed the feed within 15 min, corresponding to an oral dose of 2 × 10^6^ HAD_50_ per pig. In the wet-feed exposure model, the same amount of commercial feed (100 g) was pre-moistened with sterile water to form a porridge-like consistency before mixing with the ASFV inoculum. Because pigs did not readily consume the wet feed voluntarily, each pig in the exposed group was orally administered 100 g of ASFV-contaminated wet feed using a syringe placed at the corner of the mouth on day 0, corresponding to the same dose of 2 × 10^6^ HAD_50_ per pig. The material was delivered slowly toward the back of the oral cavity. These pigs were not sedated, and no obvious oral trauma, persistent coughing, or immediate regurgitation was observed after administration. Control pigs in both trials received the respective dry or wet feed prepared with sterile PBS instead of the virus; after the single exposure, all animals received an untreated commercial diet for the remainder of the 28-day observation period.

### ASFV-infected organ-homogenate exposure model

To model oral exposure to infected pig organ tissue, we prepared organ homogenates from an ASFV-infected donor pig. This model was intended to simulate ingestion of high-titer infected organ material that could be present in contaminated food waste. To prepare the inoculum, a 7-week-old pig was intramuscularly inoculated with 1 mL of ASFV (1 × 10^7^ HAD_50_/mL) in the neck region. The donor pig showed typical ASF clinical signs including high fever (>41 °C), anorexia, and lethargy, and died at 5 dpi. Immediately following death, necropsy was performed under sterile conditions. The spleen and liver showed splenomegaly and hepatic congestion. Twenty-five grams each of the spleen and liver (total 50 g) were aseptically collected and placed in sterile containers on ice. The tissue samples were homogenized in 100 mL of PBS to create a 33% (w/v) organ suspension. This pooled suspension was designed to mimic ingestion of mixed infected offal from naturally infected carcasses. However, because spleen and liver were pooled before titration, organ-specific viral loads in the spleen and liver were not assessed separately. The viral titer of the collected organ homogenate was determined to be 2.5 × 10^5^ HAD_50_/mL. Oral administration of 20 mL homogenate corresponded to a dose of 5 × 10^6^ HAD_50_ per pig.

Each pig in the exposed group received 20 mL of the organ homogenate via oral administration using a needle-free syringe. The homogenate was administered slowly to the back of the oral cavity to ensure complete ingestion. Control pigs received 20 mL of PBS following the same administration protocol. All exposures occurred as a single event on day 0 (considered 0 dpi), and the animals also resumed normal feeding with uncontaminated commercial diet.

### Clinical sign monitoring, sample collection, and molecular detection for ASFV DNA

Clinical signs and rectal temperatures were recorded daily for all pigs until the end of the experiment (28 dpi) or until humane endpoints were reached. Clinical sign scores were calculated based on previously established criteria (8). Rectal temperatures were measured each morning, with fever defined as a rectal temperature ≥40.5 °C. Pigs showing severe clinical scores or moribund conditions were humanely euthanized according to institutional guidelines. In the organ-homogenate trial, samples (blood, nasal, oral, and rectal swab) were collected to characterize infection kinetics at 0, 2, 4, 6, 8, and 10 dpi, or until death or a humane endpoint. In the feed trials, animals were monitored clinically each day; if high fever or ASF-compatible signs had developed, serial sampling would be initiated every 2 days. Because no pig in either feed trial developed fever, blood samples were collected at 28 dpi to assess detectable viremia. Blood samples were collected from the jugular vein, and three swab samples (nasal, oral, and rectal) were obtained using sterile cotton swabs. Each swab was placed immediately in 1 mL of PBS and vortexed for 30 seconds, and the eluate was stored at −80 °C until analysis. DNA was extracted from 200 μL of whole blood and swab eluates using the DNeasy Blood & Tissue Kit (Qiagen, Hilden, Germany) according to the manufacturer's instructions. ASFV DNA detection was performed using the VDx ASFV qPCR kit (Median Diagnostics, Chuncheon, Republic of Korea), and samples with Ct values ≤ 35 were considered positive in accordance with a previous study (8). All dead or euthanized animals were necropsied within 2 h, and the pigs that survived to 28 dpi were also necropsied at the end of the observation period.

### Statistical analysis

Statistical analyses were performed using SPSS version 29.0.2.0 (IBM Corp., Armonk, NY, USA). For experiments where viral loads were detected, the estimated clinical sign scores and Ct values from blood as well as swab samples at different time points were analyzed using repeated measures analysis of variance. Data are expressed as mean ± SD or mean ± SEM, as indicated, with *p* < 0.05 considered statistically significant.

## Results

### Clinical and virological outcomes in pigs exposed to ASFV-contaminated dry and wet feed

After a single oral exposure to ASFV-contaminated dry or wet feed, none of the pigs in either group developed clinical signs consistent with ASF during the 28-day observation period, and rectal temperatures remained within the normal range. Throughout the study, ASFV DNA was not detected in blood at 28 dpi from any pig in the dry- or wet-feed groups, indicating that no systemic infection was detected under the present experimental conditions. However, given the small sample size of the wet-feed group, these results should be interpreted as preliminary. All pigs in both feed exposure groups survived to 28 dpi without mortality (Table 1).

**Table 1:** Mortality and survival of pigs after single oral exposure to ASFV via dry feed, wet feed, or organ homogenate.

Group	*n*	Mortality rate (%)	Median survival (days)	Mean survival (days ±SD)	Days of death (range)
Dry feed	5	0	≥28	≥28	–
Wet feed	3	0	≥28	≥28	–
Organ homogenate	5	100	8	8.4 ± 1.8	6–11

### Survival, fever, and clinical signs in pigs fed ASFV-infected organ homogenate

**Figure 2:**
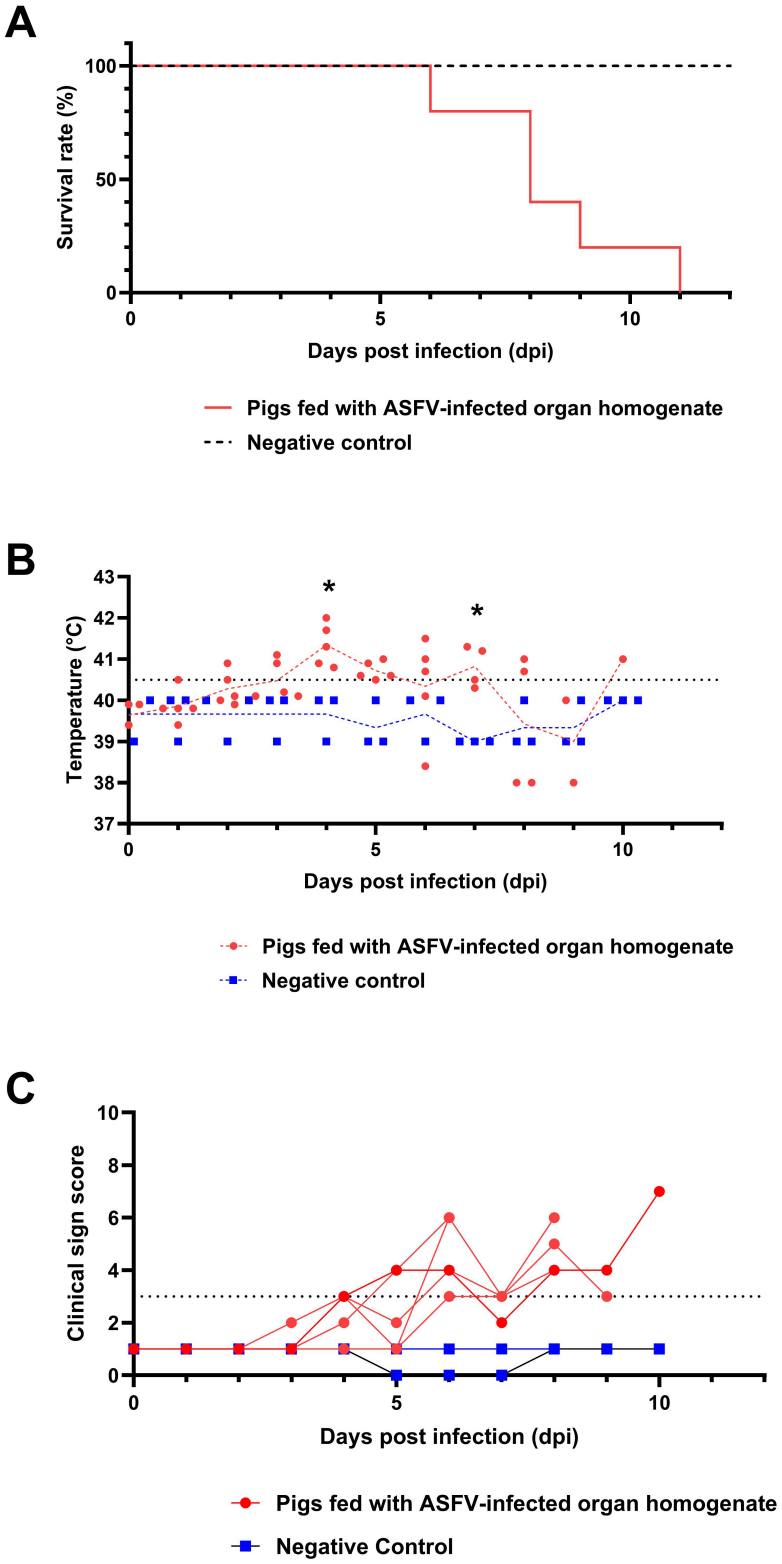
Survival and clinical responses after oral exposure to ASFV-infected organ homogenate. **(A)** Survival rates of pigs fed ASFV-infected organ homogenate (red) and negative control (black) groups. **(B)** Individual rectal temperatures of pigs fed ASFV-infected organ homogenate (red) and negative control (blue) groups. The horizontal black dotted line denotes the fever threshold (40.5 °C). *p < 0.05 (two-way ANOVA). **(C)** Time-course clinical sign scores of pigs fed ASFV-infected organ homogenate (red) and negative control (blue) groups. Clinical scores were calculated by summing subscores for fever, behavior, skin, digestive, respiratory, and body condition signs (0–3 points each; maximum 18 points per day). The horizontal black dotted line (score 3) indicates the cut-off used to define clinically affected pigs.

All pigs in the negative control group survived until the end of the experiment, whereas a single oral administration of ASFV-infected organ homogenate resulted in 100% mortality in the exposed group (Figure 2A). The first death occurred at 6 dpi, and all five pigs died by 11 dpi, consistent with an acute disease course following a single exposure (Table 1).

Rectal temperatures in negative control pigs remained within the physiological range throughout the study, while pigs in the organ homogenate group developed fever shortly after ingestion (Figure 2B). High fever (≥40.5 °C) first appeared at 3.0 ± 0.4 dpi, with mean peak rectal temperatures of 41.3 ± 0.2 °C at 4 dpi (Table 2). Group mean temperatures in the exposed group remained elevated until 7 dpi, with significantly higher values than in the negative control group at 4 and 7 dpi (two-way analysis of variance, *p* < 0.05).

**Table 2:** Viremia and ASFV shedding in organ homogenate-fed pigs (*n* = 5). Values are presented as mean ± SEM.

Parameter	Onset (dpi)	Peak value (Ct or °C)	Peak time (dpi)
Fever	3.0 ± 0.4	41.3 ± 0.2	4
Viremia	2.8 ± 0.5	14.6 ± 0.5	4
Nasal shedding	4.0 ± 0.0	25.2 ± 1.1	6
Oral shedding	3.2 ± 0.5	31.0 ± 0.4	6
Rectal shedding	4.4 ± 0.4	27.7 ± 1.1	6

Clinical scores in the negative control group remained below 1 throughout the study (Figure 2C). In contrast, pigs in the organ homogenate group first showed clinical signs at 3 dpi, and the majority exceeded the predefined clinical threshold of 3 points between 4 and 7 dpi, presenting with lethargy, reduced feed intake, and respiratory signs including labored breathing. Peak scores reached 6–7 points shortly before death or humane end-points, indicating that a single ingestion of infected organ homogenate was sufficient to induce systemic clinical disease and fatal outcomes in all exposed pigs.

### Viremia and viral shedding dynamics in pigs fed ASFV-infected organ homogenate

**Figure 3:**
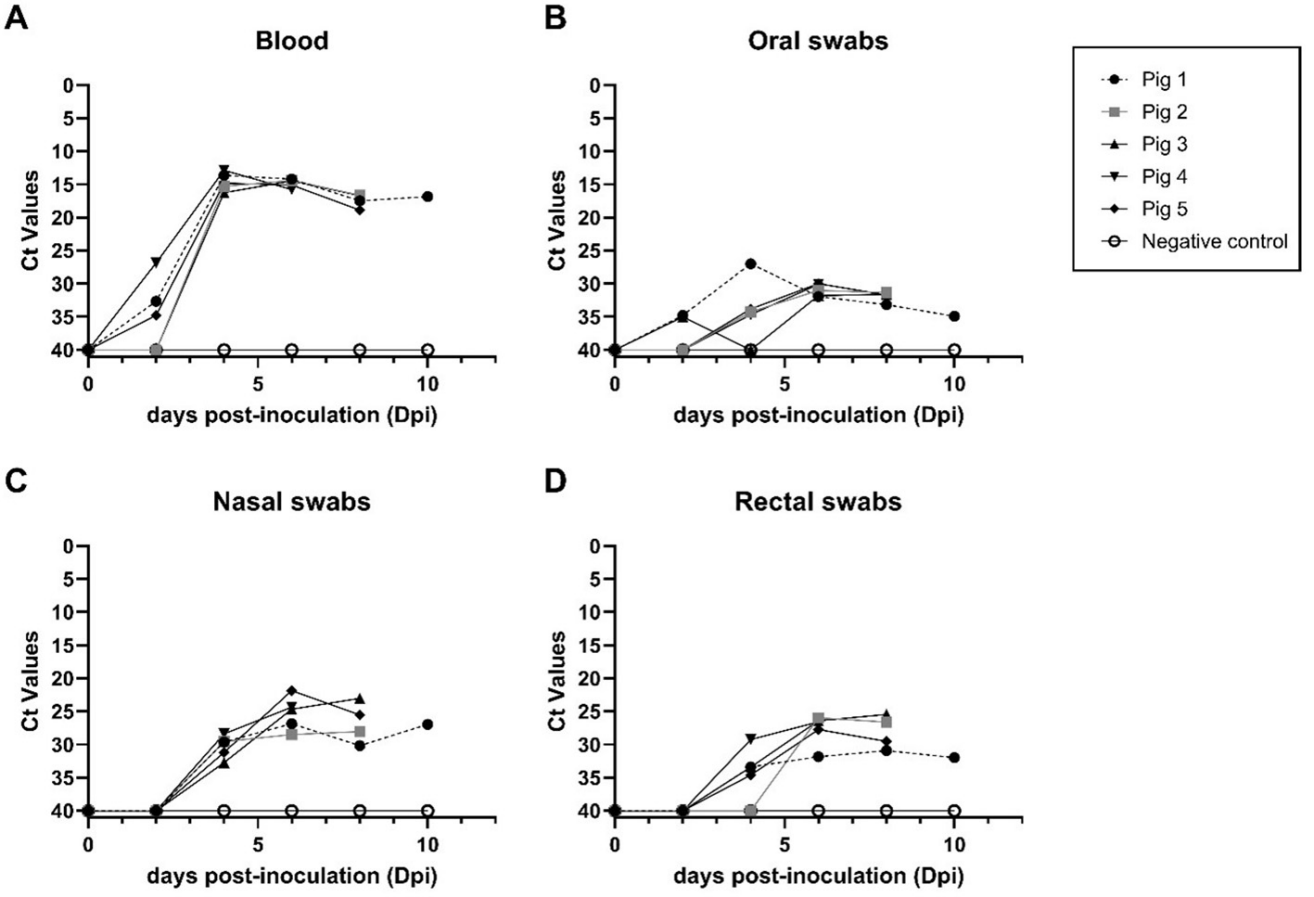
Detection of ASFV DNA in blood and swab samples from pigs fed ASFV-infected organ homogenate. **(A)** Quantitative PCR Ct values for ASFV DNA in blood collected from individual pigs at 0, 2, 4, 6, 8, and 10 days post-inoculation (dpi). **(B–D)** Quantitative PCR Ct values for ASFV DNA in oral **(B)**, nasal **(C)**, and rectal **(D)** swabs collected from the same pigs at the indicated dpi. Each symbol represents an individual pig (Pigs 1–5), and open circles indicate samples from negative-control pigs. Lower Ct values correspond to higher ASFV genome loads, and samples with Ct values ≤ 35 were considered positive.

To characterize the virological course of ASFV infection following oral exposure to infected tissue homogenate, ASFV DNA levels in blood and nasal, oral, and rectal swabs were analyzed throughout the experimental period (Figure 3 and Table 2). ASFV DNA was first detected in blood at 2.8 ± 0.5 dpi, with mean Ct values around 33, and viremia rapidly increased to peak levels at 4 dpi (mean Ct: 14.6 ± 0.5) (Figure 3A). Ct values remained low through 6 dpi and ASFV DNA was still detectable at 8 and 10 dpi in pigs that survived to these time points, demonstrating sustained systemic infection following a single oral exposure. Among swab samples, oral shedding showed the earliest onset at 3.2 ± 0.5 dpi, with the lowest Ct values at 6 dpi (mean Ct: 31.0 ± 0.4; Figure 3B and Table 2). Nasal shedding onset was at 4.0 ± 0.0 dpi, with peak viral loads at 6 dpi (mean Ct: 25.2 ± 1.1), yielding the lowest Ct values among the swab sample types (Figure 3C). Rectal shedding was the most delayed, with onset at 4.4 ± 0.4 dpi, and peaked at 6 dpi (mean Ct values of 27.7 ± 1.1) (Figure 3D). Viral DNA in rectal swabs remained detectable through 8 and 10 dpi.

### Gross pathological findings

**Figure 4:**
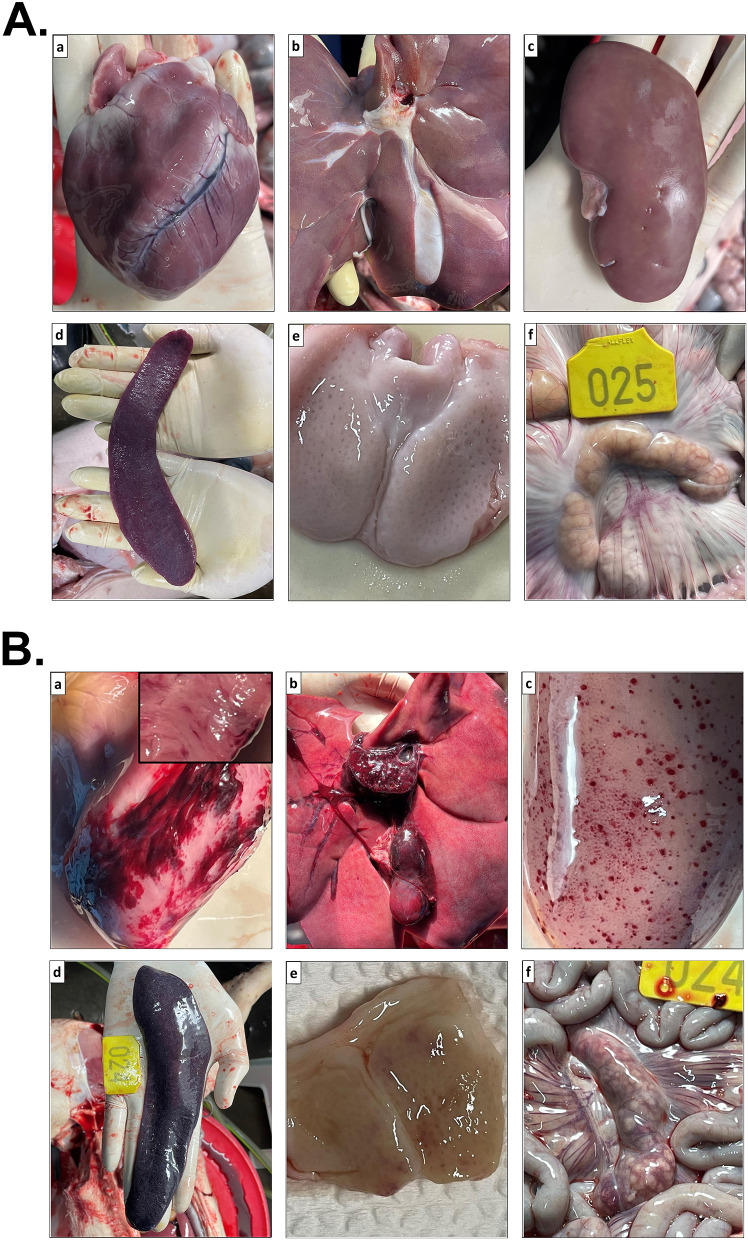
Gross lesions in pigs exposed to ASFV-contaminated wet feed and pigs fed ASFV-infected organ homogenate. **(A)** Representative gross appearance of the heart **(a)**, liver and gallbladder **(b)**, kidney **(c)**, spleen **(d)**, tonsil **(e)**, and mesenteric lymph nodes **(f)** from a pig exposed to ASFV-contaminated wet feed, showing no obvious lesions in any of the examined organs. **(B)** Representative gross lesions in a pig fed ASFV-infected organ homogenate, including myocardial hemorrhages; inset shows the cross-sectional view of the myocardium **(a)**, hepatic congestion with hemorrhages in the gallbladder wall **(b)**, renal cortical petechial hemorrhages **(c)**, hemorrhagic splenomegaly with congestion **(d)**, tonsillar hemorrhages **(e)**, and hemorrhagic enlargement of mesenteric lymph nodes **(f)**.

No gross lesions were observed in pigs from the wet- or dry-feed groups. In the wet-feed group, the heart, liver and gallbladder, kidney, spleen, tonsil, mesenteric lymph nodes, colon, and other examined organs showed no ASF-compatible lesions (Figure 4A). Similarly, no ASF-compatible macroscopic lesions were found in the dry-feed group, and their necropsy findings are not presented separately herein.

In contrast, all pigs in the organ homogenate group presented gross lesions compatible with acute ASF at necropsy (Figure 4B and Table 3). The most consistent findings were hemorrhagic lesions in the liver, gallbladder, kidneys, spleen, and lymph nodes (5/5 pigs for each site; Table 3). The liver showed diffuse congestion accompanied by hemorrhages in the gallbladder wall. The kidneys exhibited cortical petechiae, and the spleen exhibited splenomegaly. Focal to multifocal tonsillar hemorrhages and myocardial hemorrhages were each detected in 2 of 5 pigs. No gross lesions were noted in the lungs, colon, or other examined organs (Figure 4B and Table 3).

**Table 3:** Organ-specific macroscopic lesions and number of pigs affected after oral administration of ASFV-infected organ homogenate.

Organ/tissue	Typical gross lesions	No. of pigs affected (*n*/5)
Heart		Myocardial hemorrhages	2/5
Liver/gallbladder		Hepatic congestion and gallbladder hemorrhages	5/5
Kidney		Petechial hemorrhages	5/5
Spleen		Hemorrhages and congestion (± splenomegaly)	5/5
Tonsil		Focal to multifocal hemorrhages	2/5
Lymph node	Inguinal	Hemorrhages	5/5
	Mesenteric	Hemorrhages	5/5
	Submandibular	Hemorrhages	5/5

## Discussion

In this study, exposure to ASFV-contaminated feed at a nominal dose of 2 × 10^6^ HAD_50_ did not result in detectable systemic infection in either the dry-feed (*n* = 5) or wet-feed (*n* = 3) group within the 28-day observation period. No clinical signs or gross lesions were observed during the 28-day experimental period, and ASFV DNA was not detected in blood collected at 28 dpi. These findings are consistent with previous studies demonstrating the difficulty of establishing feed-mediated ASFV transmission. Niederwerder et al. (11) reported that the Georgia 2007/1 strain infected pigs via plant-based feed at a minimum infectious dose of 10^4^ 50% tissue culture infectious dose (TCID_50_), but with a median infectious dose as high as 10^6.8^ TCID_50_, suggesting that feed-mediated infection is highly dose-dependent and not reliably established even at high viral concentrations. Olesen et al. (12) further reported that only 3 (50%) of 6 pigs became infected after three oral administrations with dose at approximately 10^5^ TCID_50_, while lower doses (10^3^-10^4^ TCID_50_) failed to cause infection despite up to 13 repeated oral exposures over 28 days. In contrast, Cho et al. (15) reported 100% infection after direct intraoral inoculation of 10^6^ HAD_50_ using a syringe, bypassing the feed matrix. Although direct comparison between studies is limited by differences in viral titration methods (TCID_50_ vs. HAD_50_) and virus strains, the overall findings suggest that infection through contaminated feed was not detected under the tested conditions. Several factors could account for the absence of detectable infection in the feed groups compared with the organ-homogenate group. First, the commercial feed matrix may reduce the effective infectious dose through viral adsorption to feed particles or partial inactivation during mixing, whereas the protein- and lipid-rich organ tissue matrix is considered to provide greater environmental stability to ASFV (10, 11). Second, voluntary consumption of dry feed over 15 min limited the duration of direct contact between the virus and the tonsillar mucosa, a primary site of ASFV entry (16), whereas direct delivery of organ homogenate by syringe to the back of the oral cavity facilitated exposure of the oropharyngeal lymphoid tissues. Third, the nominal dose administered via feed (2 × 10^6^ HAD_50_) was lower than that via organ homogenate (5 × 10^6^ HAD_50_), and the residual infectious titer of the feed at the time of consumption was not measured. Therefore, the actual dose delivered to the pigs may have been lower than the nominal value. In our study, this absence of detectable infection, combined with the single-exposure design, may have placed the administered dose (2 × 10^6^ HAD_50_) near or below the practical threshold for reliably establishing ASFV infection in pigs.

While feed contamination did not produce detectable systemic infection under the conditions tested, swill feeding with contaminated pork products represents a well-documented transmission pathway for ASFV (10, 16). Accordingly, the challenge dose for the organ homogenate experiment was designed to reflect viral loads observed in naturally infected pig tissues. Sehl-Ewert et al. (17) reported viral genome concentrations ranging from 1 × 10^2^ to 9 × 10^5^ genome copies per 5 μL of blood in ASFV-infected wild boar carcasses, with organ tissues generally showing viral loads approximately one logarithmic step lower than blood samples (17, 18). A previous study also demonstrated that spleen and liver tissues from ASFV-inoculated pigs exhibited the highest viral concentrations among parenchymal organs, with mean viral loads exceeding 10^6^ genome copies per gram of tissue during acute infection (19). Although genome copy numbers do not directly equate to infectious viral titers, these data suggest that the dose used in the organ-homogenate model (5 × 10^6^ HAD_50_ per pig) falls within a biologically relevant range for naturally infected tissues.

Oral administration of ASFV-infected organ homogenate resulted in 100% mortality, with systemic viremia preceding viral shedding first via oral then nasal and rectal routes. This temporal sequence is consistent with the hematogenous dissemination pattern reported in previous ASFV transmission studies (16, 20). Our findings provide experimental evidence that ingestion of ASFV-infected pork organ homogenate, a model for high-risk tissue exposure through contaminated food waste, resulted in a markedly different outcome compared with contaminated feed under the conditions tested. The results also highlighted the need for strict enforcement of swill feeding bans and proper disposal of infected carcasses (21, 22).

To our knowledge, this is the first controlled study to directly compare the ASFV infection efficiency between contaminated feed and infected pork organ tissue consumption under standardized experimental conditions. Nevertheless, several limitations should be considered. First, the experiments compared biologically relevant exposure scenarios rather than strictly dose-matched conditions; the feed and organ-homogenate groups differed in inoculum source, nominal dose, matrix composition, and mode of oral delivery. Therefore, our findings should be interpreted as outcomes under the specific doses and matrices tested, rather than as definitive evidence that contaminated commercial feed cannot transmit ASFV. In addition, the present feed-exposure model represents extrinsic contamination of finished commercial feed and cannot be extrapolated to virus introduction through feed ingredients during the manufacturing process. Second, back-titration of the ASFV-contaminated feed at the time of administration was not performed, and water activity after mixing was not measured. Therefore, the exact amount of viable virus entering the oral cavity remains unclear, and the nominal dose may not have been equivalent to the infectious dose actually delivered. Third, pigs in the feed trials that remained clinically normal were only evaluated by blood qPCR at 28 dpi; serological testing (e.g., anti-p72 ELISA) and qPCR on tonsils, mandibular lymph nodes, and retropharyngeal lymph nodes were not performed. Therefore, transient low-level or localized infection cannot be completely excluded. Fourth, group sizes were small, particularly in the wet-feed trial (*n* = 3); therefore, the absence of detectable infection in this group should be regarded as a preliminary observation rather than definitive evidence of non-transmission. Therefore, the current data support contrasting outcomes under the tested conditions rather than a definitive quantitative ranking of all feed- vs. tissue-associated transmission risks. Future studies employing repeated-exposure protocols with larger sample sizes, defined feed matrices, and residual infectivity measurements at the time of feeding would provide more refined data for quantitative risk assessments.

In conclusion, this study provides experimental evidence that a single oral exposure to ASFV-contaminated commercial feed under our experimental conditions did not result in detectable systemic infection in pigs, whereas ingestion of ASFV-infected organ homogenate via the same route resulted in rapid systemic infection and 100% mortality. However, the potential for feed-mediated transmission under different conditions cannot be completely ruled out and warrants further investigation. These markedly different infection outcomes between the two exposure scenarios tested support continued enforcement of swill-feeding bans and safe disposal of infected carcasses. Our findings also provide a valuable experimental basis supporting biosecurity measures for ASF prevention in both ASF-endemic and ASF-free countries.

## Data Availability

The original contributions presented in the study are included in the article, further inquiries can be directed to the first and corresponding authors.
